# The BAG-1 cochaperone is a negative regulator of p73-dependent transcription

**DOI:** 10.1038/sj.bjc.6604985

**Published:** 2009-03-17

**Authors:** X-H Wang, D O'Connor, M Brimmell, G Packham

**Affiliations:** 1 Cancer Research UK Clinical Centre, Cancer Sciences Division, University of Southampton School of Medicine, Southampton, UK

**Keywords:** BAG-1, HSC70, HSP70, p73, transcription, apoptosis

## Abstract

High-level expression of Bcl-2 associated athanogene (BAG-1) protects cancer cells from stress-induced cell death and growth inhibition. These protective effects of BAG-1 are dependent on interactions with the HSC70 and HSP70 chaperones. However, the key stress-response molecules that are regulated by a BAG-1/chaperone mechanism have not been identified. In this study, we investigated the effects of BAG-1 overexpression on the function of p53 family proteins, p53, p63 and p73. Overexpression of BAG-1 isoforms interfered with the transactivating activity of p73 and p63, but had modest and variable effects on p53-dependent transcription. p73 and BAG-1 interacted in intact cells and overexpression of BAG-1 decreased the expression of p73. siRNA-mediated ablation of endogenous BAG-1 increased the activity of a p73-responsive promoter and this was reversed by knock-down of p73. The ability of BAG-1 to modulate p73 activity and expression, and to interact with p73 were dependent on amino acid residues required for the interaction of BAG-1 with HSC70 and HSP70. These results show that BAG-1 inhibits the transactivating functions of p73 and provide new insight into the mechanisms that control the expression of p73. Inhibition of p73 function may be one mechanism that contributes to the pro-survival activity of BAG-1.

Bcl-2 associated athanogene (BAG-1) is a multifunctional protein that interacts with multiple cellular targets and modulates a wide range of cellular processes (Alberti *et al*, 2003; Townsend *et al*, 2003a, 2005; Gehring, 2004). Overexpression of BAG-1 protects cells from various apoptotic stimuli, enhances proliferation and metastasis, and modulates the transcriptional activity of a variety of nuclear hormone receptors. BAG-1 is essential for the survival and differentiation of haemopoietic and neuronal cells in mice (Gotz *et al*, 2005). Functional and expression studies suggest that overexpression of BAG-1 may play an important role in diverse cancer types (Cutress *et al*, 2002; Tang, 2002; Sharp *et al*, 2004). For example, BAG-1 is frequently overexpressed in breast cancer and can correlate with important clinical parameters (Tang *et al*, 1999, 2004; Turner *et al*, 2001; Townsend *et al*, 2002; Cutress *et al*, 2003; Pusztai *et al*, 2004; Sirvent *et al*, 2004).

In human cells BAG-1 exists as three major isoforms (BAG-1S, BAG-1M and BAG-1L) derived by alternate translation initiation from a single mRNA (Figure 1). All BAG-1 isoforms contain a C-terminal, evolutionary conserved BAG domain (Takayama and Reed, 2001) and a central ubiquitin-like domain (ULD), but the larger isoforms have unique N-terminal extensions. In general, the functional significance of these variable N-terminal regions is poorly understood. However, BAG-1L possesses a nuclear localisation sequence and is a predominantly nuclear protein, whereas the other isoforms partition between the cytoplasm and nucleus (Packham *et al*, 1997; Takayama *et al*, 1998; Yang *et al*, 1998; Brimmell *et al*, 1999).

The C-terminal BAG domain is comprised of a bundle of three *α*-helices of which helices 2 and 3 mediate electrostatic interactions with the ATPase domain of the 70 kDa heat shock proteins, HSC70 and HSP70 (Briknarova *et al*, 2001; Sondermann *et al*, 2001). BAG-1 acts as a cochaperone and stimulates nucleotide exchange of HSC70/HSP70 (Hohfeld and Jentsch, 1997; Takayama *et al*, 1997; Luders *et al*, 2000b; Brehmer *et al*, 2001; Nollen *et al*, 2001). HSC70 and HSP70 play important roles in multiple cell processes, for example, by effects on protein (re)folding and degradation, and on the expression and activity of nuclear hormone receptors (Mayer and Bukau, 2005; Daugaard *et al*, 2007; Grad and Picard, 2007). Binding to these multifunctional proteins may explain, at least in part, the multiple effects associated with BAG-1 overexpression. The BAG-1 ULD is required for the interaction of BAG-1 with the proteasome (Luders *et al*, 2000a), and substantial evidence shows that BAG-1 can act to coordinate the function of chaperones and the proteasome in the degradation of specific proteins (Arndt *et al*, 2007). BAG-1 can interact simultaneously with HSC70 and the proteasome (Luders *et al*, 2000a; Alberti *et al*, 2002), and its ability to influence chaperone function may facilitate the unloading of chaperone clients in the vicinity of the proteasome to enhance degradation. BAG-1 also interacts with CHIP, an E3 ubiquitin ligase, which plays a key role in protein triage (i.e., degradation versus refolding) decisions (McDonough and Patterson, 2003). BAG-1 and CHIP cooperate to target the glucocorticoid receptor for proteasomal degradation (Demand *et al*, 2001).

Functional studies show that BAG-1 isoforms promote the survival, proliferation and metastasis of cancer cells. For example, overexpression of BAG-1S or BAG-1L increases breast cancer cell survival *in vitro* and tumour growth *in vivo* (Kudoh *et al*, 2002). Our own studies (Townsend *et al*, 2003b) showed that overexpression of BAG-1 isoforms provided robust protection from cell death and long-term growth inhibition induced by heat shock, and other cellular stress, including hypoxia, radiation and certain cytotoxic agents. RNAi-mediated knock-down of BAG-1 is also sufficient to promote apoptosis (Sawitzki *et al*, 2002; Clemo *et al*, 2008). The survival-promoting function of BAG-1 was dependent on the BAG domain as a BAG-1 mutant lacking this region failed to promote cell survival in breast cancer cells (Kudoh *et al*, 2002; Townsend *et al*, 2003b). In addition to HSC70/HSP70, the BAG domain also acts as a docking site for c-Raf; BAG-1 activates c-Raf independent of Ras (Wang *et al*, 1996; Song *et al*, 2001). However, the critical functional requirement for the BAG domain seemed to be chaperone binding as the introduction of mutations that specifically ablated HSC70/HSP70 interaction interfered with BAG-1-mediated survival in breast cancer cells (Townsend *et al*, 2003b) and other cell systems (Townsend *et al*, 2004). Thus, the survival function of BAG-1 is dependent on HSC70/HSP70. However, the specific molecular regulators of stress-induced apoptosis that are targeted by BAG-1 remain to be identified.

Members of the p53 family of transcription factors (p53, p63 and p73 and their splice variants) are critical regulators of stress-induced apoptosis (Murray-Zmijewski *et al*, 2006; McKeon and Melino, 2007; Stiewe, 2007). These proteins accumulate or are activated following cellular stress (including DNA damaging treatments and oncogenic stress) and transactivate target genes to induce cell cycle arrest (e.g., GADD45, p21^cip1^), apoptosis (e.g., Bax, Puma, IGFBP3, PIG3) or activate negative-feedback control loops (e.g., Mdm2). The function of p53 family proteins is required for normal stress-induced apoptosis (Agami *et al*, 1999; Gong *et al*, 1999; Yuan *et al*, 1999; Flores *et al*, 2002; Gressner *et al*, 2005; Stiewe, 2007) and they are frequently inactivated in cancer cells by mutations (p53 in particular) or alternate mechanisms. A number of proteins that have been shown to be overexpressed in cancer cells act, at least in part, by inhibiting the function of p53 family proteins. These proteins may be attractive therapeutic targets, as interfering with their function can lead to a reactivation of tumour suppressor function. For example, Mdm2 is overexpressed in many cancers with wild type p53 and inhibits p53 function by inhibition of transcriptional activity and targeting for degradation (Toledo and Wahl, 2006). Inhibitors of the p53:Mdm2 interaction induce p53-dependent apoptosis and are being developed as anti-cancer drugs (Dudkina and Lindsley, 2007).

Our earlier study showed that BAG-1 overexpression suppressed stress-induced apoptosis in MCF7 breast cancer cells (Townsend *et al*, 2003b), despite the presence of wild type p53, p63 and p73 in these cells (Gudas *et al*, 1995; Toh *et al*, 2005). This suggested that BAG-1 could interfere with the normal function of these proteins. As transcriptional regulation plays a major role in the function of p53, p63 and p73, we investigated the effects of BAG-1 on transactivation by p53 family proteins.

## Materials and methods

### Cell lines and culture

SaOs2 (human osteosarcoma), HEK293 (human embryonic kidney) and NIH3T3 (mouse fibroblast) cell lines were obtained from American Type Culture Collection (ATCC; Manassas, VA, USA) and maintained in Dulbecco's Modified Eagle's medium (Gibco, Paisley, UK) supplemented with 10% (v/v) foetal calf serum (FCS) (PAA Laboratories, Yeovil, UK), 1 mM l-glutamine and penicillin/streptomycin (Gibco). H1299 (human non-small lung carcinoma) cells were obtained from ATCC and maintained in RPMI 1640 medium (Gibco) supplemented with 10% (v/v) FCS, 1 mM l-glutamine and penicillin/streptomycin.

### Plasmids

The reporter plasmids Bax-lux, GADD45-luc, Mdm2-luc, Pig3-luc, IGFBP3B-luc and the p53 expression plasmid (Rowan *et al*, 1996; Hsieh *et al*, 1999) were kindly provided by Prof. Xin Lu, (Ludwig Institute for Cancer Research, Oxford, UK). The p63, p73*α* and p73*β* expression plasmids (De Laurenzi *et al*, 1998, 2000) were a kind gift of Prof. Gerry Melino (Medical Research Council, Toxicology Unit, Leicester, UK). Human BAG-1S, BAG-1M and BAG-1L isoform specific expression constructs and point mutations have been described earlier (Townsend *et al*, 2003b, 2004). pcDNA3 plasmid was from Invitrogen Life Technologies (Paisley, UK). pGL2-Basic (Promega, Southampton, UK) and the human Bcl-X IB promoter construct pBcl-XIB (MacCarthy-Morrogh *et al*, 2000) were used as control reporter plasmids.

### Transfections and reporter gene assays

For luciferase assays, SaOs2 cells (5 × 10^4^) were plated in 24-well tissue culture plate one day before transfection. Cells were transfected using FuGene 6 transfection reagents (Roche Applied Science, Burgess Hill, UK) according to the manufacturer's instructions. Empty vector pcDNA3 was used to maintain equal quantity of total DNA per transfection. At 48 h after transfection, cells were washed with cold phosphate-buffered saline (PBS), collected by centrifugation and resuspended in 100 *μ*l cell lysis buffer (0.65% (v/v) IGEPAL CA-630, 10 mM Tris(hydroxymethyl)methylamine (Tris)-HCl, 1 mM ethylenediaminetetraacetic acid (EDTA) disodium salt, 150 mM NaCl, pH 8.0) and incubated on ice for 5 min. The cell lysate was clarified by centrifugation and luciferase activity was measured using the Luciferase Assay System reagents (Promega) and a Sirius luminometer (Berthold Detection System, Oak Ridge, TN, USA). In the experiments to determine the effect of BAG-1 on p73*α* expression levels, H1299 cells were plated in 10-cm tissue culture dishes and co-transfected with 1 *μ*g of p73*α* expression construct in the presence or absence of 7 *μ*g of BAG-1S expression construct or pcDNA3. After 24 h, expression of p73*α*, BAG-1 and PCNA (loading control) were analysed by immunoblotting.

### Immunoblotting

Immunoblots were carried out as described earlier (Brimmell *et al*, 1999) using the following primary antibodies: rabbit polyclonal anti-BAG-1 (TB3, (Brimmell *et al*, 1999)), mouse monoclonal anti-BAG-1 (3.10 G3E2; (Brimmell *et al*, 1999)), mouse monoclonal anti-p73 antibody E4 (Santa Cruz Biotechnology, Santa Cruz, CA, USA), rabbit polyclonal anti-p73 antibody R26 (generated by immunisation of rabbits with purified GST-p73*α*^1−131^ fusion protein), rabbit polyclonal anti-*β*-actin antibody (Sigma, Poole, UK) and mouse monoclonal anti-PCNA antibody (Santa Cruz Biotechnology). Horseradish peroxidase-conjugated secondary antibodies were from Amersham (GE Healthcare UK, Amersham, UK) and bound immunocomplexes were detected using SuperSignal West Pico Chemiluminescent reagents (Perbioscience UK Ltd, Pierce, Northumberland, UK). To quantify the effects of overexpression of BAG-1 on p73*α* expression, immunoblots were analysed using Quantity One program (BioRad, Hemel Hempstead, UK). The expression of p73*α* was normalised to the expression of PCNA and the relative expression of p73*α* in the absence of BAG-1 overexpression was set at 1.0.

### RNA interference

Control siRNA (control 1), siRNA against Bcl-w (control 2), siRNA against human BAG-1 and siRNA against human p73 were obtained from Ambion Ltd (Huntingdon, UK) as annealed double-stranded RNA-DNA hybrids. Their sequences are: Bcl-w sense 5′-r(GCUGGAGAUGAGUUCGAGA)d(tt)-3′ and antisense 5′-r(UCUCGAACUCAUCUCCAGC)d(tg), BAG-1 sense 5′-r(GGUUGUUGAAGAGGUCAUA)d(tt)-3′ and antisense 5′-r(UAUGACCUCUUCAACAACC)d(tg)-3′, hp73 sense 5′-r(CGGAUUCCAGCAUGGACGU)d(TT)-3′ and antisense 5′-r(ACGUCCAUGCUGGAAUCCG)d(TT)-3′. H1299 cells were co-transfected with siRNA oligonucleotides at a final concentration of 75 nM, together with a reporter plasmid (pig3-luc at 400 ng) using Lipofectamine 2000 (Invitrogen) according to the manufacturer's instructions. After 72 h, cells were harvested and lysed for western blotting and luciferase assay.

### Quantitative-reverse transcription-polymerase chain reaction (Q–RT–PCR)

Total RNA was isolated using Trizol (Invitrogen) and the quantity and quality of RNA was analysed using a Agilent 2100 Bioanalyser (Agilent Technologies Inc., South Queensferry, UK). cDNA was synthesised using oligo(dT) and MMLV reverse transcriptase (Promega) according to the manufacturer's instructions. Q–RT–PCR was carried out in 20-*μ*l reactions containing 5 *μ*l cDNA, 10 *μ*l Universal Taqman PCR master mix (Applied Biosystems, Warrington, UK) and 1 *μ*l of the Taqman Gene Expression Assay of interest (Applied Biosystems). Expression assays used for this study were p73 (Hs00232088_m1) and *β*-actin (Hs99999903_m1). All reactions were carried out in duplicate using the ABI PRISM 7500 Sequence Detection System (Applied Biosystems) according to the following thermal cycle protocol: 94°C for 10 min, followed by 40 cycles at 94°C for 15 s and 60°C for 1 min. Control reactions with no cDNA were run on each plate for each Taqman gene Expression Assay used and no amplification was detected in any control reaction. All expression values were normalised using expression of *β*-actin as a control.

### Co-Immunoprecipitations

H1299 cells were transfected (FuGene 6) in 4 × 100 mm tissue culture plates, each with 5 *μ*g of p73*α* expression plasmid together with 5 *μ*g of BAG-1S expression plasmids, or 5 *μ*g of pcDNA3 empty vector. Cells were washed and harvested in cold PBS and resuspended in 1.5 ml of HMKEN buffer (10 mM *n*-2-hydroxyethylpiperazine-*N′*-2-ethanesulfonic acid (pH 7.2), 5 mM MgCl_2_, 142 mM KCl, 2 mM ethylene glycol-bis(b-aminoethylether)*N,N,N′,N′*-tetra-acetic acid, 0.2% (v/v) IGEPAL, and Protease Inhibitor Cocktail (Sigma)) by passing repeatedly through 21-gauge and 25-gauge needles, followed by incubation on ice for 30 min. The lysate was clarified by centrifugation. A portion (50 *μ*l) of the resultant cell lysate was retained as a whole cell lysate. The remaining sample was pre-cleared using protein G-Sepharose beads (pre-blocked with 5% (w/v) skimmed milk overnight) for 30 min at 4°C on a Spiramixer. Protein G-Sepharose beads were removed by centrifugation. To immunoprecipitate BAG-1, the lysate was divided into two parts and incubated with the BAG-1-specific rabbit polyclonal antibody TB3 or with pre-immune control serum (both at 4 *μ*l/500 *μ*l lysate), respectively. After overnight incubation at 4°C, the lysate was incubated with protein G-Sepharose beads at 4°C for 4 h, and immunocomplexes were removed by centrifugation. The beads were washed four times using HMKEN buffer, re-suspended in 50 *μ*l SDS-PAGE sample buffer and heated at 95°C for 5 min before immunoblot analysis.

## Results

### BAG-1 isoforms interfere with the transactivating function of p73*α*

**Figure 2:**
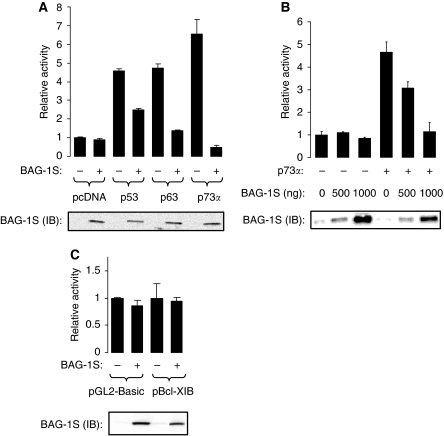
Effect of Bcl-2 associated athanogene (BAG-1)S overexpression on transcriptional regulation by p53 family proteins. (**A**) SaOs2 cells were transfected with 100 ng of Bax-luc reporter construct and 100 ng of p53, 100 ng of p63 or 50 ng of p73*α* expression plasmids, respectively, in the presence or absence of BAG-1S expression construct (2500 ng). Transfected cells were analysed for luciferase activity (top) and BAG-1S expression (by immunoblotting (IB); bottom) 48 h after transfection. Data shown are the mean luciferase activity (±s.d.) of duplicate transfections normalised to cells transfected with Bax-luc only (set to 1.0). (**B**) SaOs2 cells were transfected with 100 ng of Bax-luc reporter construct in the absence or presence of p73*α* expression plasmid (50 ng) and increasing amount of BAG-1S expression construct (0, 500, 1000 ng). Transfected cells were analysed for luciferase activity (top) and BAG-1S expression (bottom) 48 h after transfection. Data shown are the mean luciferase activity (±s.d.) of duplicate transfections normalised to cells transfected with Bax-luc only (set to 1.0). (**C**) SaOs2 cells were transfected with 200 ng of pGL-Basic or Bcl-XIB reporter constructs in the absence or presence of 1000 ng of BAG-1S expression plasmid and luciferase activity (top), and BAG-1S expression (by immunoblotting (IB); bottom) was measured after 48 h. Data shown are the mean luciferase activity (±s.d.) of duplicate transfections normalised to cells without BAG-1S overexpression (set to 1.0). The experiments shown in (**A**–**C**) are representative of two similar experiments.

We carried out transient transfection assays to determine whether the major BAG-1 isoform, BAG-1S, modulates the transcriptional activities of p53 family proteins. p53-null SaOs2 cells were selected for this study as they have been widely used for investigations of p53 family protein function (Jost *et al*, 1997; Mantovani *et al*, 2004; Melino *et al*, 2004). SaOs2 cells were transfected with a Bax promoter–reporter construct and p53, p63 or p73*α* expression plasmids, in the presence or absence of a human BAG-1S expression plasmid (Figure 2A). BAG-1S overexpression did not have a significant effect on the basal expression of the Bax promoter but did interfere with the ability of p53, p63 and p73*α* to increase promoter expression. Whereas the ability of BAG-1S to modulate p53 function was modest and variable between experiments (mean inhibition from two separate experiments each carried out in duplicate (±s.d.) was 22±33%), p63 and p73*α* functions were strongly inhibited by BAG-1S (mean inhibition 76±7% and 91±1%, respectively). Co-expression of p53, p63 or p73*α* did not significantly alter the levels of BAG-1S (Figure 2A).

**Figure 3:**
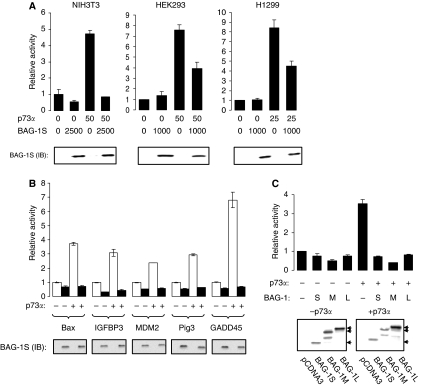
Regulation of p73*α* transcriptional activity by Bcl-2 associated athanogene (BAG-1) isoforms. (**A**) Human embryonic kidney (HEK)293, NIH3T3 and H1299 cells were transfected with the Bax-luc reporter (100 ng) and the indicated amounts (ng) of p73*α* and BAG-1S expression plasmids. Luciferase activity (top) and BAG-1S expression (by immunoblotting (IB); bottom) was measured after 48 h. Data shown are the mean luciferase activity (±s.d.) of duplicate transfections normalised to cells transfected with Bax-luc only (set to 1.0). Experiments shown are representative of at least three similar experiments. (**B**) SaOs2 cells were transfected with various promoter–reporter constructs with or without p73*α* (25 ng for transfections with the Pig3 promoter, 50 ng for transfections with the Bax promoter, IGFBP3 or MDM-2 promoter or 100 ng for transfections with the GADD45*β* promoter) and in the presence (closed bars) or absence (open bars) of BAG-1S (1000 ng). The following amounts of reporter constructs were used in each transfection; Bax, 100 ng; GADD45*β*, 200 ng; IGFBP3, 50 ng; MDM-2, 50 ng; or Pig3, 100 ng. Luciferase activity (top) and BAG-1S expression (by immunoblotting (IB); bottom) was measured after 48 h. Data shown are the mean luciferase activity (±s.d.) of duplicate transfections normalised to cells transfected with each reporter construct in the absence of BAG-1S or p73*α* (set to 1.0). Experiments shown are representative of at least two similar experiments. (**C**) SaOs2 cells were transfected with the Bax-luc reporter construct (100 ng) and the p73*α* expression plasmid (50 ng) and BAG-1S, BAG-1M or BAG-1L expression plasmids (1000 ng), as indicated. Luciferase activity (top panel) was measured after 48 h. Data shown are the mean luciferase activity (±s.d.) of duplicate transfections normalised to cells transfected with the Bax reporter construct in the absence of BAG-1 or p73*α* (set to 1.0). Experiment shown is representative of four similar experiments. The bottom panel shows BAG-1 protein expression in transfected cells analysed by immunoblotting. Arrows indicate the BAG-1L, BAG-1M and BAG-1S isoforms. Note that some faster migrating BAG-1 forms are detected, especially in cells transfected with the BAG-1M expression plasmid. These may derive from internal translation initiation or degradation (Lee *et al*, 2007).

As the effects of BAG-1S on p73*α* function were most dramatic, we focused our analysis on this interaction. Plasmid-titration experiments showed that the effects of BAG-1S overexpression were concentration dependent (Figure 2B) and were specific, because BAG-1S overexpression did not interfere with the activity of control promoters not regulated by p73*α* (Figure 2C). The inhibitory effects of BAG-1S were also observed in all the cell lines tested (HEK293, NIH3T3 and H1299; Figure 3A) and using a range of p73-responsive promoter constructs (IGFBP3, GADD45, Pig3 and MDM-2; Figure 3B). All three BAG-1 isoforms inhibited p73*α*-mediated transcription when overexpressed in SaOs2 cells (Figure 3C). The expression of BAG-1 isoforms was not altered by co-expression of p73*α*.

### BAG-1S is a more effective inhibitor of p73*α* compared with p73*β*

**Figure 4:**
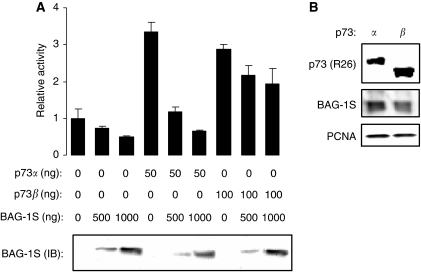
Effect of Bcl-2 associated athanogene (BAG-1) S on the activity of p73*α* and p73*β*. SaOs2 cells were transfected with Bax-luc reporter construct (100 ng) and the indicated amounts of p73*α*, p73*β* and BAG-1S expression constructs. After 48 h luciferase activity was measured (top) and BAG-1 expression analysed by immunoblotting (bottom). In (**A**), data shown are the mean luciferase activity (±s.d.) of duplicate transfections normalised to cells transfected with the Bax reporter construct in the absence of BAG-1 or p73*α* (set to 1.0). Experiment shown is representative of three similar experiments. (**B**) SaOs2 cells were transfected with BAG-1S (500 ng), p73*α* (50 ng) or p73*β* (100 ng) expression constructs and analysed by immunoblottting using the R26 antibody. BAG-1S and PCNA were analysed as controls.

p73 is expresssed as multiple isoforms (Murray-Zmijewski *et al*, 2006). p73*β* is generated by alternative splicing and has a truncated C-terminus compared with p73*α*. p73*β* is transcriptionally active and we therefore compared the ability of BAG-1S to inhibit transcriptional activation by p73*α* and p73*β* (Figure 4A). Whereas BAG-1S overexpression substantially reduced p73*α*-mediated transcription (mean inhibition 84±5%, mean derived from three identical experiments, ±s.d.), the activity of p73*β* was relatively modestly affected (mean inhibition 32±9%). The difference between the effects of BAG-1S on p73*α* and p73*β* was statistically significant (Student's *t*-test, *P*=0.003). p73 isoforms were expressed at approximately equivalent levels (Figure 4B). In these experiments, we used 100 ng of p73*β* expression plasmid (compared with 50 ng of p73*α* expression plasmid) to achieve approximately equivalent levels of activation of the Bax reporter construct. However, BAG-1S also failed to effectively repress p73*β* activity when cells were co-transfected with 50 ng of p73*β* expression plasmid (data not shown).

### BAG-1 knockdown reactivates p73 function

**Figure 5:**
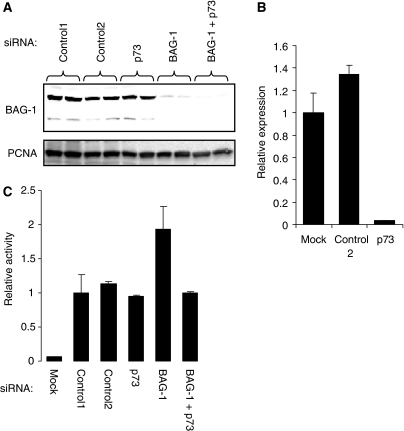
Effect of Bcl-2 associated athanogene (BAG-1) knock-down on endogenous p73 activity. H1299 cells were co-transfected in duplicate with control, p73 or BAG-1-specific siRNAs and the Pig3-luc promoter–reporter plasmid (400 ng). BAG-1 protein levels (**A**) and luciferase activity (**C**) were analysed 72 h after transfection. (**C**) shows mean luciferase activity (±s.d.) of duplicate transfections normalised to luciferase activity in cells transfected with control 1 siRNA (set to 1.0). Mock-transfected cells are the cells without any siRNA/reporter plasmid. In (**A**), PCNA expression was also analysed as a loading control. Experiment shown is representative of three similar experiments. (**B**) H1299 cells were transfected with control or p73-specific siRNAs, or mock transfected in the absence of any siRNAs, and expression of p73 RNA was analysed by quantitative-reverse transcription (Q–RT)–PCR after 72 h.

As overexpression studies showed that BAG-1 inhibited the transcription-activating function of p73, we used RNA interference to determine whether similar functional interactions occur between endogenous BAG-1 and p73 proteins. H1299 cells were selected for these studies because of the very high efficiency of siRNA-mediated knock-down obtained. H1299 cells were transfected with the Pig3 promoter–reporter construct to monitor p73 activity and BAG-1 and p73 were depleted by siRNA. The BAG-1 siRNA has been validated in earlier studies (Clemo *et al*, 2008), and immunoblot analysis confirmed the effective knock-down of BAG-1 expression in H1299 cells (94±6% reduction in BAG-1 siRNA transfected cells, mean±s.d. derived from four experiments), which predominantly express BAG-1 L (Figure 5A). Because of the absence of suitable antibodies to reliably detect endogenous p73, we were unable to confirm knockdown of p73 at the protein level. However, this siRNA has been validated in earlier studies (Basu *et al*, 2003) and Q-RT-PCR analysis showed a clear knock-down of p73 RNA (Figure 5B). Depletion of BAG-1 resulted in a 1.9±0.3 fold increase in the activity of the Pig3 promoter (mean±s.d. of four experiments), compared with cells transfected with control siRNA (Figure 5C). Knock-down of p73 reversed the activation of the Pig3 promoter observed in cells transfected with the BAG-1 siRNA, but had no effect on Pig3 promoter activity when tested alone (Figure 5C).

### Inhibition of p73*α* function by BAG-1S requires helix 2 and 3 of the BAG domain

**Figure 6:**
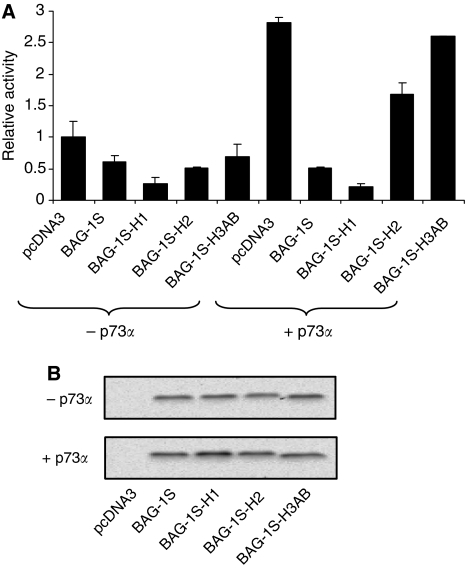
Effect of Bcl-2 associated athanogene (BAG-1) C-terminal point mutations on p73*α* activity. SaOs2 cells were transfected with 100 ng of Bax-luc reporter construct and p73*α* expression plasmid (100 ng) and wild type/mutant BAG-1S expression constructs (2500 ng) as indicated. After 48 h luciferase activity was measured (**A**) and BAG-1 expression analysed by immunoblotting (**B**). In (**A**), data shown are the mean luciferase activity (±s.d.) of duplicate transfections normalised to cells transfected with the Bax reporter construct in the absence of BAG-1 or p73*α* (set to 1.0). Experiment shown is each representative of three similar experiments.

We showed earlier that suppression of apoptosis by BAG-1S requires amino acids within helix 2 and 3 of the BAG domain important for interaction with HSC70/HSP70 (Townsend *et al*, 2003b, 2004). We therefore analysed the effect of BAG-1 C-terminal mutations on the ability of BAG-1S to inhibit p73*α* activity. Mutations within helix 2 or 3, in BAG-1S-H2 (Q169A, K172A) and BAG-1S-H3AB (Q201A, D208A, Q212A) significantly reduced the ability of BAG-1S to inhibit p73*α*-mediated transcription (Figure 6A). By contrast, mutations within helix 1 in BAG-1S-H1 (E112A and K116A) did not interfere with p73*α*-mediated transcription. Immunoblot analysis showed that the wild type and mutant BAG-1S proteins were expressed at approximately equivalent levels (Figure 6B). As shown earlier (Briknarova *et al*, 2001; Townsend *et al*, 2004; Lee *et al*, 2007), mutation of helix 2 and 3, but not helix 1, prevented interaction of BAG-1S with HSC70 in co-immunoprecipitation assays (Figure 8A).

### BAG-1S interacts with p73*α* and decreases p73*α* expression levels

**Figure 7:**
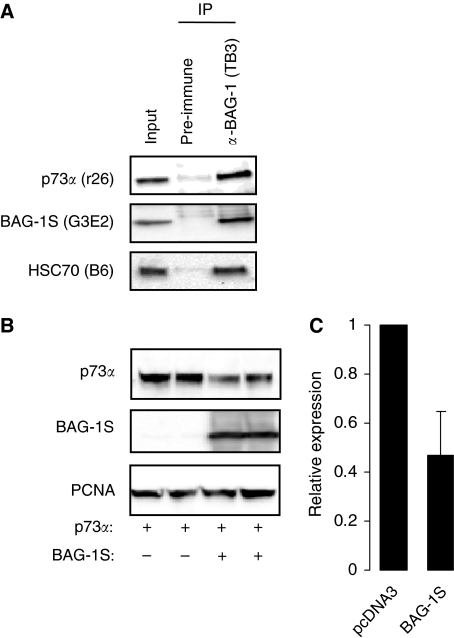
Bcl-2 associated athanogene (BAG-1) S interacts with p73*α* and decreases the expression levels of co-expressed p73*α*. (**A**) H1299 cells were transfected with p73*α* and BAG-1S expression constructs. After 24 h, immunoprecipitations were carried out using the BAG-1-specific TB3 antibody or pre-immune serum as a control. Immunoprecipitates were analysed by immunoblotting using antibodies specific for p73 (R26), BAG-1 (G3E2) or HSC70 (B6). ‘Input’ is the lysate from transfected cells before immunoprecipitation. (**B**) H1299 cells were transfected with a p73*α* expression construct in the presence or absence of a BAG-1S expression construct. After 24 h, expression of p73*α*, BAG-1S and PCNA (loading control) were analysed by immunoblotting. (**C**) Quantitation of p73*α* expression in BAG-1S and control (pcDNA3 transfected) cells. The expression of p73*α* in control cell lysates was set to 1.0. Data are mean (±s.d.) expression levels derived from eight similar experiments.

We carried out immunoprecipitation experiments to determine whether BAG-1S and p73*α* associate in intact cells. H1299 cells were transfected with BAG-1S and p73*α* expression plasmids, and BAG-1 complexes immunoprecipitated. In addition to the expected association with HSC70, there was a clear interaction between BAG-1S and p73*α* (Figure 7A). We also determined whether overexpression of BAG-1S altered the levels of p73*α*. In H1299 cells co-overexpressing BAG-1S, the levels of p73*α* were significantly reduced (Figure 7B). On an average, co-expression of BAG-1S reduced p73*α* expression by ∼50% (mean of eight experiments; Student's *t*-test, *P*=6 × 10^−5^) compared with control cells (Figure 7C). Therefore, when overexpressed in cells, BAG-1S and p73*α* interact and BAG-1S expression leads to a reduction in p73*α* levels.

### p73*α* binding and regulation of expression is dependent on helix 2 and 3 of the BAG-1S BAG domain

**Figure 8:**
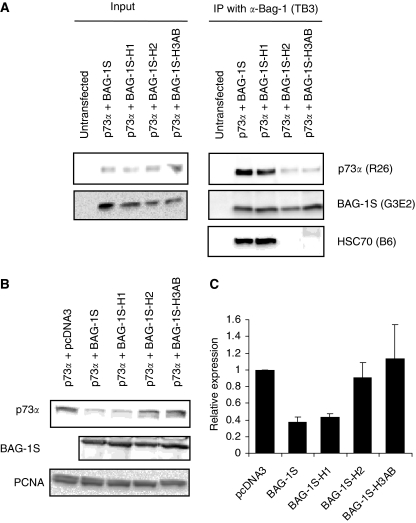
Effect of Bcl-2 associated athanogene (BAG-1) C-terminal point mutations on p73*α* interaction and modulation of p73*α* expression levels. (**A**) H1299 cells were transfected with the indicated p73*α* and wild type/mutant BAG-1S expression constructs. After 24 h, immunoprecipitations were carried out using the BAG-1-specific TB3 antibody. Immunoprecipitates were analysed by immunoblotting using antibodies specific for p73 (R26), BAG-1 (G3E2) or HSC70 (B6). ‘Input’ is the lysate from transfected cells before immunoprecipitation. (**B**) H1299 cells were transfected with the p73*α* expression construct in the presence or absence of wild type/mutant BAG-1S expression constructs. After 24 h, expression of p73*α*, BAG-1S and PCNA (loading control) were analysed by immunoblotting. (**C**) Quantitation of p73*α* expression in wild type and mutant BAG-1S transfected cells. The expression of p73*α* in control cell lysates was set to 1.0. Data are mean (±s.d.) expression levels derived from two identical experiments.

As regulation of p73*α* activity is dependent on residues within helix 2 and 3 of the BAG domain, we analysed the effects of these mutations on the ability of BAG-1S to interact with p73*α* and to decrease the levels of p73*α*. Mutations within helix 2 and 3, but not helix 1, decreased the interaction between BAG-1S and p73*α* (Figure 8A) and reduced the ability of BAG-1S to decrease the expression of p73*α* (Figures 8B and C). Therefore the ability of BAG-1S to inhibit p73*α* activity, to interact with p73*α* and to decrease p73*α* expression levels are all dependent on the residues with helix 2 and 3 of the BAG-1S BAG domain. As these residues are also required for interaction with HSC70/HSP70, chaperones are likely to be critical mediators of the regulation of p73*α* by BAG-1S.

## Discussion

Earlier study has shown that BAG-1 contributes to the inappropriate survival of malignant cells. However, the specific molecular targets of BAG-1 that mediate survival remain to be identified. Although originally identified as a Bcl-2 interacting protein (Takayama *et al*, 1995) the significance of this interaction remains unclear and at present data showing a role for Bcl-2 modulation in BAG-1-mediated survival are lacking. We have shown earlier that endogenous BAG-1 enhances the function of NF-*κ*B in colorectal cancer cells (Clemo *et al*, 2008). BAG-1 also interacts with and interferes with the function of the stress-responsive, pro-apoptotic GADD34 protein (Hung *et al*, 2003). However, the requirements for cochaperone binding in these activities are not clear.

Data presented here show that overexpression of BAG-1 inhibits the transcriptional activating functions of p53 family proteins (p73 in particular) and this may represent one mechanism by which BAG-1 interferes with stress-induced apoptosis. The absence of robust reagents to reliably detect endogenous p73 forced us to focus on overexpression studies, and this is one of the major limitations of our study. However siRNA-mediated ablation showed similar functional interactions between endogenous BAG-1 and p73 because BAG-1 knock-down leads to an increase in p73-dependent transcription. p73 is activated in response to a variety of chemotherapeutic drugs and *γ*-irradiation, and is an important determinant of cellular sensitivity to apoptosis (Agami *et al*, 1999; Gong *et al*, 1999; Yuan *et al*, 1999; Flores *et al*, 2002; Bergamaschi *et al*, 2003). Although p73 is rarely mutated in cancer cells, the expression of alternate spliced isoforms (e.g., ΔNp73) might interfere with p73 function in a dominant negative manner (Melino *et al*, 2002; Murray-Zmijewski *et al*, 2006). p73 activity can also be regulated by interaction with several cellular partners such as Mdm-2, Yap, ASPP family proteins and these are often altered in cancer cells. Our data suggest that overexpression of BAG-1 might be an additional mechanism that limits p73 function in malignant cells.

The inhibitory function of BAG-1 was more pronounced for p73*α*, compared with p73*β*, a splice variant that is transcriptionally active and is frequently co-expressed in cancer cells. Compared with p73*α*, p73*β* lacks a C-terminal SAM domain. The molecular function of the SAM domain is not known, but is considered to act as a negative control domain (Liu and Chen, 2005). It is interesting that, p63 also contains an N-terminal SAM domain whereas p53 does not. Thus, the ability of BAG-1 to modulate p53-family proteins may involve, but not absolutely require, the SAM domain. Although the function of the SAM domain is likely to be multifunctional, including effects of co-activator recruitment (Liu and Chen, 2005) and DNA binding, our data further suggest that the SAM domain may also act to confer chaperone-dependent negative regulation. However, it is important to note that under our experimental conditions we did not detect enhanced transcriptional activation by p73*β*, compared with p73*α*, as shown earlier (De Laurenzi *et al*, 1998; Ueda *et al*, 2001; Liu and Chen, 2005).

The ability of BAG-1S to inhibit p73*α* function seems to be mediated through physical association. Both the ability of BAG-1S to modulate p73*α*-transcriptional activity and to associate with p73*α* was dependent on specific residues within the BAG-1 BAG domain that are also required for binding to HSC70 and for promotion of cell survival (Townsend *et al*, 2004; Lee *et al*, 2007). The data are consistent with a model in which the binding of BAG-1 to p73*α* is mediated by HSC70/HSP70, but studies with purified components are required to test this. One consequence of formation of this complex may be a reduction in the steady state levels of p73*α*, as co-expression of BAG-1S and p73*α* reduced p73*α* levels by ∼50%. Decreased expression of p73*α* is not an artefact of co-expression (e.g., competition for transcription/translation machinery), as certain mutants of BAG-1S did not show this activity although expressed at equivalent levels. Moreover, this function of BAG-1S was dependent on residues required for binding to HSC70, again strongly implicating chaperones in this effect. One mechanism by which BAG-1S may decrease p73*α* function is by increasing proteolytic degradation. However, the decrease in p73 levels (50%) did not fully account for the inhibition of p73*α* activity observed in transfection studies (80%). Thus, alternate mechanisms are also likely to contribute, and chaperone-dependent changes in the conformation of p73 and its association with co-regulatory molecules may also be important.

A key question is to what extent the ability of BAG-1S to decrease p73*α* levels is dependent on the ability of BAG-1 to coordinate the activity of the proteasome and chaperones in protein triage decisions (Arndt *et al*, 2007). We have not shown that the reduction in p73*α* levels are proteasome-mediated, but there is substantial evidence that p73*α* levels can be controlled by ubiquitination and proteasomal degradation. Several E3-ligase have been shown to modulate p73 turnover, including Itch and UFD2a (Hosoda *et al*, 2005; Oberst *et al*, 2005; Rossi *et al*, 2005). Proteasome binding of BAG-1 is dependent on the ULD (Luders *et al*, 2000a; Demand *et al*, 2001). Our analysis of BAG-1 ULD mutants did not clarify the role of this domain in controlling p73 function because deletion of the entire ULD or mutation of a conserved lysine (K80 in BAG-1S) required for cell survival (Townsend *et al*, 2003b) resulted in significant destabilisation and stabilisation of BAG-1S, respectively, making interpretation of data obtained for these proteins unclear.

Although BAG-1 very effectively inhibited p73 function, its effects on p53 were modest and variable between experiments. These results are probably consistent with those of Matsuzawa *et al* who showed that BAG-1S did not interfere with p53-dependent transcription in HEK293 cells (Matsuzawa *et al*, 1998). However, in addition to the direct effects of BAG-1 on p73-transactivating function that we have described, it remains possible that BAG-1 inhibits p53 function independent of effects in transcription. BAG-1 participates in complexes with wild type and mutant p53 (King *et al*, 2001), and although the functional implications of BAG-1 are not known, CHIP targets p53 for proteasomal degradation (Esser *et al*, 2005). BAG-1 overexpression interferes with p53-induced growth arrest and apoptosis, perhaps by interfering with downstream effector molecules, such as Siah (Danen-van Oorschot *et al*, 1997; Matsuzawa *et al*, 1998). Thus, we believe that the inhibitory effects of BAG-1 on p53 family proteins as a whole are likely to involve both direct modulation of transactivating function and indirect effects. However, further study is required to dissect the molecular details of these interactions.

## Figures and Tables

**Figure 1:**
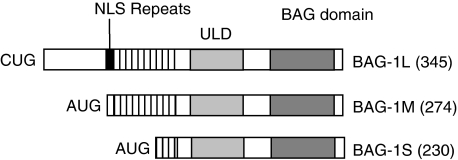
Human Bcl-2 associated athanogene (BAG-1) isoforms. The structures of the three major human BAG-1 isoforms are shown, along with their size (amino acid residues). Translation of BAG-1L initiates at an upstream CUG codon, whereas BAG-1M and BAG-1S are AUG-derived. The position of the nuclear localisation sequence (NLS), acidic repeats, ubiquitin-like domain (ULD) and BAG domain are shown.
